# A New Biomarkers Feature Pattern Consisting of TNF-****α****, IL-10, and IL-8 for Blood Stasis Syndrome with Myocardial Ischemia

**DOI:** 10.1155/2013/130702

**Published:** 2013-11-25

**Authors:** Shuzhen Guo, Jianxin Chen, Wenjing Chuo, Lei Liu, Xuanchao Feng, Hongjian Lian, Lei Zheng, Yong Wang, Hua Xie, Liangtao Luo, Chenglong Zheng, Bangze Fu, Wei Wang

**Affiliations:** ^1^School of Preclinical Medicine, Beijing University of Chinese Medicine, Beijing 100029, China; ^2^Department of Health Care, General Military Hospital of Beijing PLA, Beijing 100700, China

## Abstract

*Objective*. To explore new diagnostic patterns for syndromes to overcome the insufficiency of obtainable macrocharacteristics and specific biomarkers. *Methods*. Chinese miniswines were subjected to Ameroid constrictor, placed around the proximal left anterior descending branch. On the 4th week, macrocharacteristics, coronary angiography, echocardiography, and hemorheology indices were detected for diagnosis. IL-1, IL-6, IL-8, IL-10, TNF-**α**, and hsCRP in serum were detected, and Decision Tree was built. *Results*. According to current official-issued standard, model animals matched the diagnosis of blood stasis syndrome with myocardial ischemia based on findings, including >90% occlusion, attenuated left ventricular segmental motion, dark red or purple tongues, and higher blood viscosity. Significant decrease of IL-10 and increase of TNF-**α** were found in model animals. However, in the Decision Tree, besides IL-10 and TNF-**α**, IL-8 helped to increase the accuracy of classification to 86%. *Conclusions*. The Decision Tree building with TNF-**α**, IL-10, and IL-8 is helpful for the diagnosis of blood stasis syndrome in myocardial ischemia animals. What is more is that our data set up a new path to the differentiation of syndrome by feature patterns consisting of multiple biomarkers not only for animals but also for patients. We believe that it will contribute to the standardization and international application of syndromes.

## 1. Introduction

Treatments based on syndrome differentiation are essential to Traditional Chinese Medicine (TCM). Syndromes were named from different aspects and always including information of property or location of the disease, such as blood stasis syndrome and Qi deficiency in heart. It is widely accepted that disease and syndrome were the different aspects to the same pathological changes of a patient. When we focused on a particular disease, syndromes of TCM worked more like the criteria to further divide patients into different subtypes. These subtypes were the aims of Chinese herbal recipe and acupuncture for a long history. These subtypes were differentiated according to the major pathogenesis, and the latter was concluded from a group of symptoms and signs which are called macrocharacteristics. For the collecting of macrocharacteristics, looking, listening, question, and feeling the pulse were considered as the main methods.

Different from TCM, western medicine traditionally focused on the general character of a disease and the standardized treatment for all patients. But recently, it began to pay more attention to the differences between patients and look for the causes from genomics. Thus, the new strategy which emphasized on the diversification of treatment according to patients' gene background was known as personalized medicine. However, macrocharacters or pathologic changes are mostly decided by the function of multiple genes instead of single one. What is more, genotypes may give some clues of the differences of patients, but acquired factors contributed a lot to the phenotypes as well. Differentiating by macrocharacteristics will still be one of the most efficient ways to subtype patients for clinical purpose.

Experimental animals are the most important tool of translational medicine as well as TCM. The only difference is that the syndrome of animals should be taken into account to meet the therapeutic principles of herbal recipes besides diseases. However, most of those macrocharacteristics for patients' syndrome differentiation are difficult to get from animals. Standards and easily accessible biomarkers are desperately needed to overcome this huge gap between humans and experimental animals. Moreover, due to their poor specificities and/or sensitivities, many biomarkers for syndromes differentiation were discovered and then abandoned when used singly in the past decades. Considering the facts that a group of symptoms and signs together was used for patients' diagnosis, we believe that some kind of feature patterns consisting of several biomarkers will work better than single one.

Currently, coronary artery disease is one of the most widespread diseases, and blood stasis syndrome is the most common subtype of it. Blood stasis syndrome always performs as dark-purple tongue, pain in a fixed position, stagnated blood under skin, astringent pulse, angiemphraxis, abnormal hemorheology indices, and so forth.

Previous researches had shown that inflammation was one of the most important path physiological processes involved in blood stasis syndrome with coronary artery disease or myocardial ischemia [1, 2]. In this study, we try to explore feature patterns of blood stasis syndrome by screening biomarkers from the most easily detected and widely reported inflammation related cytokines, including interleukin-1 (IL-1), interleukin-6 (IL-6), interleukin-8 (IL-8), interleukin-10 (IL-10), tumor necrosis factor-a (TNF-*α*), and high-sensitivity C-reactive protein (hsCRP).

## 2. Material and Methods

This study was reviewed and approved by Fuwai Hospital Animal Care and Use Committee and met the criteria of the National Institutes of Health for animal research. The surgery and model evaluation were conducted in Animal Research Center at Fuwai Cardiovascular Hospital, and laboratory analysis was conducted in our laboratory.

### 2.1. Surgery

In this study, Chinese miniswines from Animal Breeding Center of China Agricultural University (Beijing, China), weighing between 20 and 30 kg, were included. Anesthesia was induced with ketamine intramuscularly and maintained by diazepam and ketamine together. Chronic myocardial ischemia was produced by placing an Ameroid constrictor (Research Instruments, SW) around the proximal left anterior descending branch (LAD) after left thoracotomy. Thoracotomy without Ameroid constrictor placing was performed in sham group. 4 weeks later, examination and evaluation were performed.

### 2.2. Observation of Macrocharacteristics

According to a scale for macrocharacteristics of swine as we reported previously, details of animals' macrocharacteristics were observed in the morning [3]. Without any disturbance, overall performances of animals were observed, including general state, activity, daily food intake, daily water intake, and urine and stool property. And a video was recorded for further analysis. Then, animals were fixed by experienced breeders in a way veterinarian commonly used. After animal calmed down, its tongue, nose, and skin of fore and hind hoofs were observed, and the description of these characteristics was written down. Hereafter, photos of these areas were taken in the natural light by a camera (Nikon D70, Japan), and Casmatch Color Card (BEAR Medic Corporation, Japan) was used as reference. To standardize the condition of photos, the same angle and same distance were set and photos were taken by the same researcher.

### 2.3. Echocardiography

In the 4th week after surgery, transthoracic echocardiography was performed with standard left ventricular short axis and apical four chamber views under anesthesia (Agilent SONOS 5500, USA). Left ventricular volumes at the ends of systole and diastole, ejection factor (EF), fractional shortening (FS), and the wall motion index were calculated according to the method previously described by the American Society of Echocardiography [4].

### 2.4. Coronary Angiography

Four weeks after surgery, selective left coronary angiography was performed (Cardicav GE OEC 9800, USA) under the guidance of manufactory to document coronary flow to the LAD. Then TIMI of LAD was analyzed.

### 2.5. Hemorheology Indices

Whole blood was collected on the 28th day after surgery from the femoral vein. Analyses of hemorheology indices were performed in 6 hours after blood collection. High shear specific viscosity and low shear specific viscosity of whole blood and reduced blood, plasma specific viscosity, hematocrit, erythrocyte deformation index, and erythrocyte aggregation index were determined by using a rotary whole blood viscometer and plasma viscometer (LG-R-80 and LG-B-190, China) in the Laboratory of Dongzhimen hospital (Beijing, China). All measurements were carried out at 37°C according to the international guidelines for the measurements of hemorheologic parameters [5].

### 2.6. ELISA Analysis

Whole blood was collected on the 28th day after surgery from the femoral vein. Serum samples were collected, and concentrations of IL-1, IL-6, IL-8, IL-10, TNF-*α*, and hsCRP were measured by enzyme-linked immunosorbent assay (ELISA) (Kangyuan Ruide Biological Technology Co., Ltd, China) according to the manufacturer's instructions.

### 2.7. Statistical Analysis

All continuous data are expressed by mean ± SEM. Independent sample *t*-test was used to compare the means between groups. All statistical analyses were performed by using SPSS 17.0 for Windows. And *P* < 0.05 was considered as significant.

### 2.8. Classification by Decision Tree

The program of Decision Tree in SPSS 17.0 was used as the classification method. IL-1, IL-6, IL-8, IL-10, TNF-*α*, and hsCRP were included as independent variables, while blood stasis syndrome as dependent variable. CRT was selected as growing method. Minimum Cases in parent and children Node were set to 10 and 5, respectively. 3-folds crossvalidation was used. 

## 3. Results

25 animals alive in 4th week were used in further analysis, including 15 animals for model group and 10 for sham group. These 10 sham-operated animals were used in the diagnosis of blood stasis syndrome, including analysis of angiography, echocardiography, macrocharacteristics, and hemorheology indices. Since no difference was found between sham-operated animals and healthy ones, for the sake of increasing specificity, we used 18 health animals together with 10 sham-operated animals in the control group when we analyzed the inflammation related cytokines and built the Decision Tree.

### 3.1. Coronary Angiography

**Figure 1:**
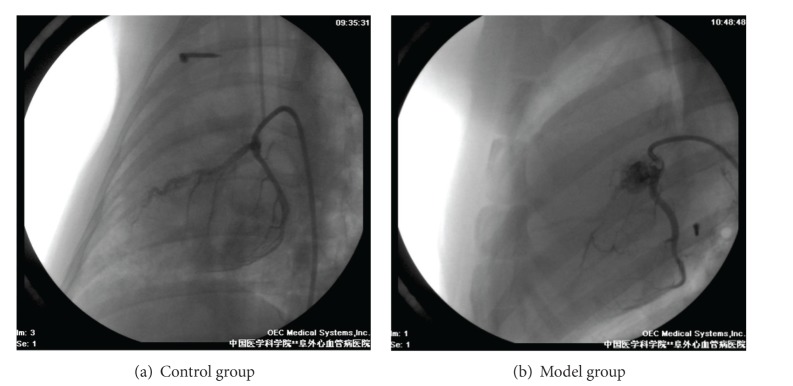
Coronary angiography.

Ex vivo angiography showed that more than 90% occlusion or even complete block of the left anterior descending artery by the Ameroid was found in model animals in the 4th week after surgery. TIMI flow grade I or II was observed in most of the model animals. There was good filling in the left anterior descending artery with TIMI flow grade III in Sham-operated animals. Results were shown in Figure 1.

### 3.2. Echocardiography

The structure and function of left ventricular were evaluated by echocardiography. Compared with sham operated animals, there was great increase of left ventricular volume and diameter at both end-systolic and end-diastolic volume (*P* < 0.01) in model animals. Anterior wall thickness of both papillary muscle and apex level was decreased at end-diastolic and end-systolic in model animals (*P* < 0.01). Interventricular septal depth was decreased at end-diastolic and end-systolic in model animals (*P* < 0.05 and *P* < 0.01). Meanwhile, papillary muscles level and the apex of the left ventricular anterior systolic wall thickening decreased (*P* < 0.05 and *P* < 0.01, resp.). In addition, septal thickness at the end-systolic decreased (*P* < 0.05). All the results above showed a segmental dysfunction of the left ventricular. There were no significant changes in ejection faction (EF) and fraction of shortening (FS), which suggested that cardiac function was still in compensatory period. Results were given in Table 1.

### 3.3. Observation of Macrocharacteristics

Model animals showed mental stress, irritation, fear, violent behaviors, strong self-defense, disorderly fur lack of luster, slightly dark red or purple tongue, and so forth, while sham-operated animals' performances gradually returned to normal, with increased appetite, neat and shiny fur, and light red or light white tongue.

### 3.4. Hemorheology Indices

As data displayed in Table 2, the whole blood viscosity and reduced blood viscosity of model animals increased significantly at a shear rate of middle (1/38) and low (1/5) (*P* < 0.05) when compared with sham-operated animals. No other changes were observed.

### 3.5. Changes of Inflammation Related Cytokines

In model group, IL-10 was decreased and TNF-*α* was increased dramatically (*P* < 0.01). No significant changes were found in IL-1, IL-6, IL-8, and hsCRP. Data were summarized in Table 3.

### 3.6. Decision Tree Building with Inflammation Related Cytokines

**Figure 2:**
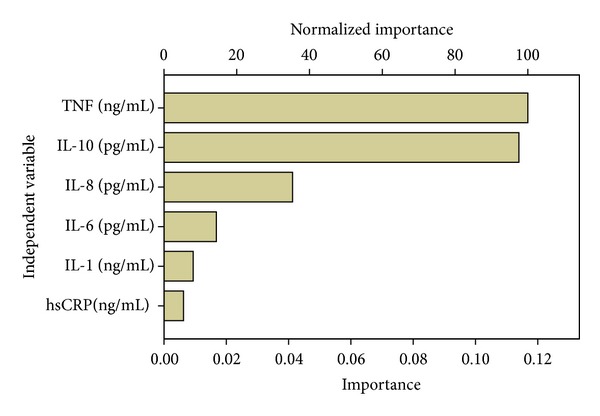
Importance of inflammation related cytokines in the Decision Tree of blood stasis syndrome. Growing method: CRT. Dependent variable: blood stasis in heart.

**Figure 3:**
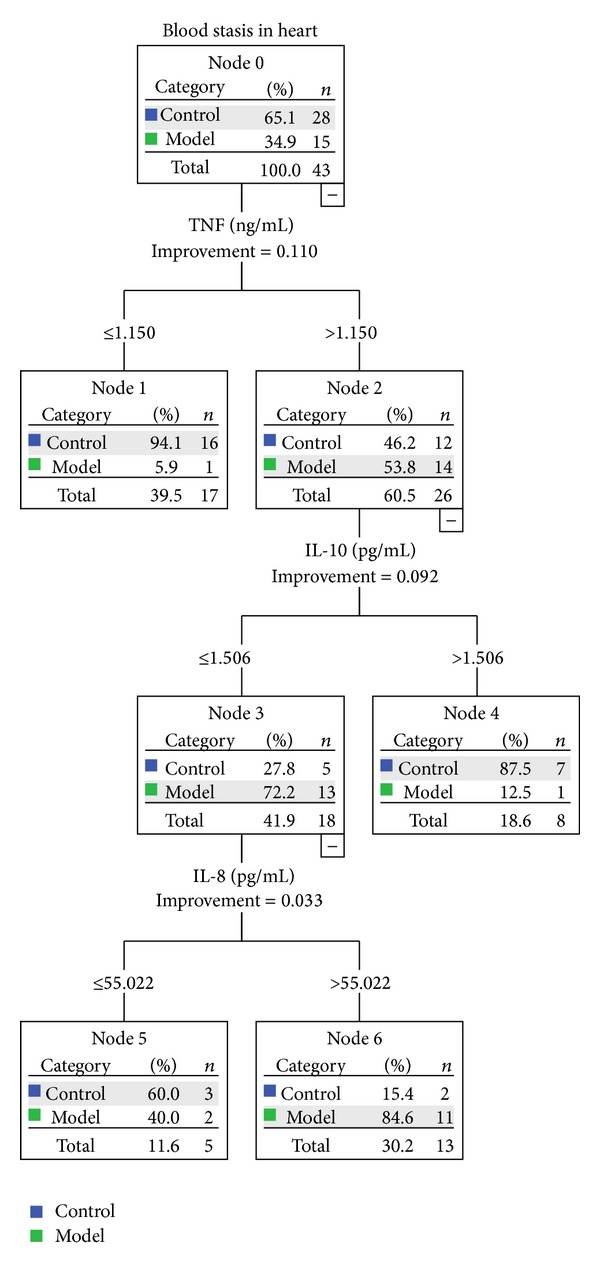
Decision Tree of blood stasis syndrome building by inflammation related cytokines.

Results were depicted in Figures 2 and 3. The number of nodes was 7, number of terminal nodes was 4, and the depth of tree was 3. TNF-*α* and IL-10 were two of the most important parameters for the blood stasis syndrome classification (improvement = 0.110 and 0.092, resp.). IL-8 takes some part of responsibility of this classification (improvement = 0.033) unless there were no big differences between two groups when compared by *t*-test. IL-1, IL-6, and hsCRP were not as important as other parameters above. The first node of classification was TNF-*α*. Most of the animals with lower than 1.150 ng/mL TNF-*α* were control group (16/17). And not only model animals (14/26) but also part of control animals (12/26) shared a higher TNF-*α* concentration (>0.115 ng/mL). Then IL-10 was used as the second node; higher IL-10 (>1.506 pg/mL) most frequently appeared in control group (7/8). When TNF-*α* was higher than 0.115 ng/mL and IL-10 was less than 1.506 pg/mL, IL-8 turned up as the third classification node; most model animals were assigned to >55.022 pg/mL group (11/13). When TNF-*α*, IL-10, and IL-8 were used together as classification nodes, the total predicted accuracy was 86%. Accuracy was much higher in control group (92.9%) than in model group (73.7%) (see Table 4).

## 4. Discussion

### 4.1. Evaluation of Myocardial Ischemia

Ameroid constrictor is a widely used instrument in myocardial ischemia models. It results in chronic ischemia due to gradual occlusion. In our study, in the 4th week after surgery, more than 90% occlusion or even complete block of the left anterior descending artery was confirmed by angiography. Significant changes were found in the structure of left ventricle and its anterior wall, such as LVEDd, LVEDs, ESV, EDV, LVAWTs, and LVAWTd at both papillary muscles and apex level. Segmental motion deficiency was found at both papillary muscles and apex level, but no big difference was found in EF or FS. At this time of point, model animals met the criteria of myocardial ischemia. These results were similar to other reports [6].

### 4.2. Differentiation of Blood Stasis Syndrome

This was the most controversial and difficult part of this study. Traditionally, syndromes were differentiated by symptoms and signs in clinic. Most parts of them were difficult to obtain from animals. Even if some macrocharacteristics were obtained from animals, diagnostic values have to be elucidated carefully by comparing their similarity with patients. In this study we used the principles we had published previously [3]. Comprehensive observation of macrocharacteristics was explored at multiple time points to reduce bias. Since there was no officially-issued standard for animal blood stasis syndrome, we used the diagnostic criteria of blood stasis syndrome issued by the Chinese Association of Integrative Medicine as the reference [7]. And according to the similarity of clinic property, the macrocharacteristics of patients in the criteria were translated to animals ones carefully. Different from the traditional blood stasis syndrome diagnostic criteria, biological indices were included as diagnostic items. Although relative changes instead of testing threshold were used for biological indices, it was still a big progress during the history of syndromes' objectification and standardization. Besides purple tongue and other macrocharacteristics, occlusion of blood vessels and changes of hemorheology indices were both considered as characters of blood stasis syndrome. Due to this big progress, differentiation of animals' syndrome began to be more feasible. Animals in the 4th week after surgery were found with angiemphraxis in coronary artery and dark purple tongue, which met 2 of the major defining characteristics and abnormal hemorheological indices, which met one of the laboratory evidences. According to these criteria, the chronic myocardial ischemia swine we established met the diagnosis of blood stasis syndrome, which was consistent with our previous report [8]. 

### 4.3. Exploring a Method of Syndrome Differentiation for Individual Animal Models

In clinic, patients were diagnosed individually based on symptoms and signs as well as inspection items with testing threshold. However, the comparison between model and control group was the only element used in the syndrome differentiation due to the shortage of diagnostic researches in animals. Moreover, even sharing the most pure genetic background, individual animal performed differently to the same treatment in the window of both macrocharacteristics and biomarkers. Thus, some samples have to be excluded to get more accurate results in many conditions. As the result, we believed that it will be better to perform individual differentiation instead of group statistic test in animal syndrome diagnosis. Since available animal macrocharacteristics are still limited, objective diagnostic items and their testing threshold were desperately needed.

A lot of researches suggested that syndrome was complicated and difficult to be distinguished by single biomarkers efficiently. It was widely accepted that a group of specific symptoms and signs are essential for the particular syndrome differentiation. We believe that a group of biological biomarkers working together may improve the sensitivity and specialty of syndrome differentiation. Nonetheless, fewer biomarkers with higher accuracy will be more efficient. What's more, for the sake of practical consideration, simple methods with good repeatability were always chosen for diagnosis use. That is to say, the balance had to be made between accuracy and practice.

Decision Tree was one of the best methods to classify samples according to several independent variables. It cannot only select the most important variables for classification but also provide the cutoff of every independent variable.

When we were seeking the possible biomarkers from multiple pathophysiological processes involved in blood stasis syndrome with myocardial ischemia, inflammation related cytokines came to our sight. A lot of researches showed that inflammation was one of the most important changes in blood stasis syndrome. Meanwhile, there are many inflammation related cytokines which were related to ischemia and/or blood stasis syndrome. Interleukin-1 (IL-1) was considered as one of the most patent prototypic inflammatory cytokines with adverse effects [9]. On the contrary, IL-10 and IL-8 played positive roles in left ventricular dysfunction and remodeling [10, 11]. Elevated IL-6 and CRP were both considered as biomarkers for adverse cardiovascular events [12, 13]. Interestingly, TNF-*α* played an ambivalent (detrimental versus beneficial) role depending on its dose and/or time [14]. And it was reported that some of these inflammation-related cytokines were involved in blood stasis syndrome [15]. All these inflammation related cytokines may be the most hopeful candidates for the biomarkers pattern.

We try to explore whether several parameters together will be better for blood stasis syndrome differentiation. And here we identified that higher TNF-*α* and lower IL-10 were closely related to it. Both TNF-*α* and IL-10 played the most important role in the Decision Tree. Interestingly, IL-8 helped to increase the accuracy of differentiation which did not change dramatically in model animals. It may become a new trend to discover biomarkers of syndromes as a feature pattern instead of single gold biomarker. Moreover, ELISA was a simple and reproductive method which is widely used in most labs, while serum is an easily obtained material from both animal and human. It is certain that after this feature pattern is validated in larger samples, it may not only become a new method for diagnosis of blood stasis with myocardial ischemia animals but also be helpful for objective diagnosis of blood stasis syndromes in patients.

## 5. Limitations

Due to the similarity of its heart with human, we chose mini swine in this research. But it was too expensive and difficult to raise many swines at the same time. If more samples were used, our findings may be more accurate and valuable for future usage.

## 6. Conclusions

We explored a new pattern of biomarkers for blood stasis syndrome with myocardial ischemia, which was a Decision Tree building with TNF-*α*, IL-10, and IL-8. This feature pattern may not only help the syndromes differentiation of individual animals but also provide a new window of syndrome diagnosis in clinic. Feature pattern may become a new hopeful way for externalizing and standardizing of the diagnosis of syndromes in traditional Chinese Medicine. Further large scale trial is needed before it becomes a new diagnostic method of animal models or patients.

## Figures and Tables

**Table 1:** Changes of heart function evaluated by echocardiography (Mean ± SEM).

	Control (*N* = 10)	Model (*N* = 15)	*P* value
LVEDd (cm)	3.208 ± 0.124**	3.895 ± 0.076	0.000
LVEDs (cm)	2.041 ± 0.043**	2.657 ± 0.072	0.000
ESV (mL)	8.559 ± 0.940**	16.115 ± 1.217	0.003
EDV (mL)	22.542 ± 2.117**	36.371 ± 1.628	0.000
IVSDs (cm)	1.185 ± 0.199**	0.833 ± 0.032	0.004
IVSDd (cm)	0.692 ± 0.029*	0.592 ± 0.020	0.024
LVAWTs (cm)			
Mitral valve level	1.029 ± 0.055	1.004 ± 0.027	0.665
Papillary muscles level	1.153 ± 0.032**	0.849 ± 0.044	0.001
Apex level	1.145 ± 0.045**	0.623 ± 0.044	0.000
LVAWTd (cm)			
Mitral valve level	0.728 ± 0.025	0.739 ± 0.017	0.754
Papillary muscles level	0.794 ± 0.034*	0.648 ± 0.027	0.012
Apex level	0.808 ± 0.040**	0.546 ± 0.032	0.000
Wall thickening (%)			
Mitral valve level	41.748 ± 6.016	36.317 ± 2.498	0.350
Papillary muscles level	47.325 ± 7.038	30.136 ± 3.268	0.023
Apex level	42.564 ± 3.194**	12.072 ± 1.887	0.000
FS (%)	35.818 ± 1.752	30.835 ± 2.780	0.372
EF (%)	61.770 ± 2.067	56.299 ± 2.020	0.189

Model versus Control group, *P < 0.05; **P < 0.01.

LVEDd: left ventricular end-diastolic dimension; LVEDs: left ventricular end-systolic dimension; ESV: end-systolic volume; EDV: end-diastolic volume; IVSDs: interventricular end-systolic septal depth; IVSDd: interventricular end-diastolic septal depth; LVAWTs: left ventricular end systolic anterior wall thickness; LVAWTd: left ventricular end diastolic anterior wall thickness; FS: fractional shortening; EF: ejection fraction.

**Table 2:** Hemorheology indices (Mean ± SEM).

	Control (*N* = 10)	Model (*N* = 15)	*P* value
Whole blood viscosity 1/150 (mPa.s)	5.224 ± 0.149	5.275 ± 0.091	0.795
Whole blood viscosity 1/38 (mPa.s)	8.758 ± 0.234	9.563 ± 0.163*	0.025
Whole blood viscosity 1/10 (mPa.s)	3.727 ± 0.112	3.608 ± 0.068	0.395
Whole blood viscosity 1/5 (mPa.s)	12.590 ± 0.362	13.987 ± 0.253*	0.012
Reduced viscosity 1/150 (mPa.s)	10.091 ± 0.300	10.438 ± 0.191	0.350
Reduced viscosity 1/38 (mPa.s)	18.861 ± 0.600	20.593 ± 0.385*	0.026
Reduced viscosity 1/10 (mPa.s)	6.189 ± 0.189	6.272 ± 0.128	0.725
Reduced viscosity 1/5 (mPa.s)	28.835 ± 0.921	31.328 ± 0.621*	0.047
Plasma viscosity (mPa.s)	1.843 ± 0.054	1.899 ± 0.042	0.465
Hematocrit (%)	43.545 ± 0.928	42.727 ± 0.717	0.550
Erythrocyte aggregation index	2.651 ± 0.069	2.534 ± 0.042	0.163
Aggregation index of integral area	498.500 ± 15.307	498.500 ± 8.502	1.000
Erythrocyte deformability index	0.367 ± 0.007	0.382 ± 0.006	0.208
Deformation index of integral area	191.800 ± 5.736	190.848 ± 3.289	0.889

Model versus Control group, *P < 0.05; **P < 0.01.

**Table 3:** Changes of inflammation related cytokines (Mean ± SEM).

	Control (*N* = 28)	Model (*N* = 15)	*P* value
IL-1 (ng/mL)	0.279 ± 0.021	0.308 ± 0.036	0.466
IL-6 (pg/mL)	288.037 ± 15.161	307.662 ± 27.806	0.542
IL-8 (pg/mL)	97.436 ± 6.673	86.832 ± 10.958	0.387
IL-10 (pg/mL)	1.467 ± 0.129	0.962 ± 0.142*	0.018
TNF-*α* (ng/mL)	1.266 ± 0.117	1.769 ± 0.147*	0.012
hsCRP (ng/mL)	10.591 ± 0.839	12.936 ± 1.324	0.126

Model versus Control group, *P < 0.05; **P < 0.01.

**Table 4:** Prediction accuracy of the Decision Tree.

Observed	Predicted		
	Control	Model	Percent correct
Control	26	2	92.9%
Model	4	11	73.3%
Overall percentage	69.8%	30.2%	86.0%

## Nonstandard Abbreviations and Acronyms

IL-1: Interleukin-1
IL-6: Interleukin-6
IL-8: Interleukin-8
IL-10: Interleukin-10
TNF-*α*: Tumor necrosis factor
hsCRP: High super C reactive protein
LAD: Left anterior descending branch
ELISA: Enzyme-linked immunosorbent assay
LVAWTd: Left ventricular end diastolic anterior wall thickness
LVAWTs: Left ventricular end systolic anterior wall thickness
LVEDd: Left ventricular end-diastolic dimension
LVEDs: Left ventricular end-systolic dimension
IVSDs: Interventricular end-systolic septal depth
IVSDd: Interventricular end-diastolic septal depth
EDV: End-diastolic volume
ESV: End- systolic volume
EF: Ejection fraction
FS: Fractional shortening.
